# Train the trainers: maintaining integrity

**DOI:** 10.1186/1940-0640-8-S1-A37

**Published:** 2013-09-04

**Authors:** Sarah Jones

**Affiliations:** 1 Public Health Wales, Cardiff, UK

## 

Public Health Wales runs a successful brief intervention training programme. Over 3000 people have been trained to deliver alcohol brief interventions across Wales by the small (2 man) central Public Health Wales alcohol team. However, the team cannot keep up with demand from the 7 Welsh Health Boards to receive training. In order to address this, a train-the-trainers course has been developed, piloted and evaluated. The evaluation was based on both post-course assessment of the delivery and has been followed up with qualitative field work to ascertain if the standards of the Public Health Wales team are being replicated by the trainees. The evaluation was targeted both at the Public Health Wales central team in regard to their train the trainers course but also in regard to the delegates ability to take the theory into practice. The key issue has been the commitment of the individuals to address the issue of alcohol as a problem across Wales. It is also apparent that those who attend the training course need to meet a number of key pre-requisites which make them better placed and more capable of delivering a brief intervention training course that will inspire others to conduct interventions. Public Health Wales was able to use the evaluation to adjust the training to make it more impactful but also to develop a list of key traits which prospective attendees would have to meet in order to effectively deliver brief interventions as part of their regular role.

